# Sex differences in physical activity, psychosocial determinants, and symptom outcomes in knee osteoarthritis: a cross-sectional analysis of a Hispanic/Latino-predominant cohort

**DOI:** 10.1007/s00296-026-06148-7

**Published:** 2026-05-23

**Authors:** Jacob Jahn, Ryan C. Rizk, Levi M. Travis, Eric Kholodovsky, Navin Kaushal, Donya Nemati, Thomas M. Best

**Affiliations:** 1 University of Miami Miller School of Medicine, 1600 NW 10th Ave, Miami, FL 33136 USA; 2 Department of Orthopedics, University of Miami, Miami, FL 33124 USA; 3 UHealth Sports Medicine Institute, University of Miami, Miami, FL 33124 USA; 4 Department of Health Sciences, School of Health & Human Sciences, Indiana University, Indianapolis, IN USA; 5 College of Nursing, The Ohio State University, Columbus, OH USA; 6 Kirwan Institute for the Study of Race and Ethnicity, Columbus, OH USA

**Keywords:** Osteoarthritis, Knee, Exercise, Self Efficacy, Pain Catastrophizing, Sex Characteristics, Psychosocial Factors, Physical Activity, Social Support, Functional Limitation, Behavioral Determinants

## Abstract

Knee osteoarthritis (KOA) is a chronic disease that often leads to persistent pain, functional limitations, and diminished quality of life. Physical activity can be an effective countermeasure but its adherence typically relies on behavioral determinants. Currently, there is limited work investigating how clinical symptoms among patients with KOA affect behavioral determinants, and there is a need for further insight into why females engage in less physical activity. The purpose of this study was to investigate these questions in a sample of patients with KOA. Patients (n = 736; mean age 56.4 years) with symptomatic KOA (Kellgren–Lawrence grades 0–4) were evaluated at a tertiary sports medicine clinic and completed validated measures of physical activity behavior and behavioral determinants (attitudes, exercise self-efficacy, social support, barriers), in addition to pain and symptoms. Physical activity score was inversely associated with pain intensity (VAS; β = −0.037, p= .004), pain catastrophizing (PCS; β = −0.124, p = .004), and functional limitation (WOMAC; β = −0.364, p < .001). Greater social support was associated with lower WOMAC (β = -.30, p < .001), lower VAS (β = -.14, p =.021), and lower PCS scores (β = -.18, p = .002). Higher exercise self-efficacy was independently associated with reduced pain catastrophizing (PCS; β = −.061, p = .017) and functional limitation (WOMAC; β = −.267, p < .001). Worsening functional status (WOMAC) was significantly associated with more negative affective attitudes (β = .19, p = .001), lower exercise self-efficacy (β = .34, p < .001), and greater perceived barriers (β = .16, p = .002). Exercise intention was a significant predictor of physical activity (β = .36, p <.001), and was itself predicted by affective attitude, social support, self-efficacy, and perceived barriers. Sex comparisons revealed that males scored significantly higher than females in physical activity (p = .015), exercise intention (p = .031), exercise self-efficacy (p = .007), and social support (p = .038), with no significant differences in affective attitude or perceived barriers. Males also reported lower symptom severity than females across VAS (p = .006), PCS (p = .007), and WOMAC (p < .001); however, sex differences in VAS were no longer significant after covariate adjustment.**Conclusions.** Higher physical activity levels were associated with lower pain and improved functionality in patients with KOA. Sex-based discrepancies in exercise self-efficacy, social support, and exercise intention could help explain why females with KOA participate in less physical activity compared with their male counterparts.

**Supplementary Information:**

The online version contains supplementary material available at 10.1007/s00296-026-06148-7.

## Introduction

Knee osteoarthritis (KOA) is a chronic, progressive musculoskeletal disease typically characterized by degeneration of articular cartilage, subchondral bone changes, and synovial inflammation [1]. KOA is among the leading causes of disability in older adults, and its global burden continues to rise with aging populations and increasing obesity prevalence [2]. As such, KOA presents a significant public health concern with tremendous personal and societal burden necessitating more personalized prevention and treatment strategies.

One of the challenges of promoting exercise among individuals with KOA is that the symptoms, specifically joint pain and stiffness, function as behavior deterrents [3]. Consequently, the limited or absence of exercise engagement is associated with muscle weakness, reduced joint stability, and increased Body Mass Index (BMI), which are in turn associated with worsening KOA symptoms [4], thereby making sustained physical activity more difficult. This pattern contributes to a self-reinforcing loop of deconditioning and functional decline, often complicated by psychological barriers such as fear of movement and reduced confidence in one’s physical abilities [5]. While exercise is widely regarded as a first-line cost-effective strategy for KOA, facilitating long-term adherence remains a challenge and is frequently attributed to increased pain, low self-efficacy, and minimal social support, both of which were investigated in our current study [6]. Although many studies have demonstrated that increased physical activity can reduce pain and improve functionality in KOA [7], psychosocial factors play a key role in determining the likelihood of successful behavior adoption and adherence. This suggests that exercise may not be uniformly beneficial for all patients and that pain-related cognitive and psychosocial factors may influence the relationship between physical activity and symptom severity.

Specifically, psychosocial variables such as affective attitude (one’s emotional response to exercise), perceived barriers to exercise (factors that hinder participation such as pain or lack of time), social support for exercise (encouragement from family, peers, and healthcare providers), and exercise self-efficacy (confidence in one’s ability to exercise regularly) are recognized as important correlates of exercise engagement and effectiveness. Notably, higher exercise self-efficacy has been shown to enhance adherence, whereas negative affective attitudes and low social support are associated with poorer outcomes [8, 9].

Another discrepancy that warrants investigation are sex discrepancies in physical activity participation, specifically females with KOA engage in less physical activity compared to their male counterparts [10]. The discrepancies in behavior could stem from behavioral determinants, symptoms, pain and functionality [11, 12]. Understanding these differences could identify sex-specific targets to address discrepancy in physical activity participation. The primary purpose of this study was to investigate if identified behavioral determinants from other population groups also contribute to explaining physical activity participation, notably affective attitude, perceived barriers, social support, self-efficacy, and exercise intention, along with KOA symptom outcomes: pain intensity (VAS), pain catastrophizing (PCS), and physical function. The secondary objective was to assess sex-based differences in exercise-related psychosocial variables to determine whether patterns of physical activity engagement and related attitudes differed between male and female patients.

## Methods

### Participants and procedure

The research protocol received approval from the University Institutional Review Board (IRB #20190389; approved July 10, 2023). Written informed consent was obtained from all participants prior to enrollment, in the participant’s preferred language (English or Spanish). A total of 736 participants were recruited at a tertiary medical center in Miami-Dade County, Florida. Eligible patients were those attending their first clinic visit, aged 18 years or older, with valid insurance, and presenting with knee pain and/or stiffness. A diagnosis of knee osteoarthritis (KOA) was established using patient history, physical examination, and radiographic evaluation, including anteroposterior, lateral, and Merchant (skyline) views. Radiographic severity was graded by a sports medicine physician blinded to study details, applying the Kellgren-Lawrence (KL) classification system. The anatomical site of OA (tibiofemoral and/or patellofemoral) was also documented. After obtaining informed consent in the participant’s preferred language (English or Spanish), one of three trained interviewers administered standardized questionnaires in the corresponding language. The methodology employed in this study was adapted from our previously published work [13].

### Measures

#### Demographic and clinical variables

During the clinic visit, participants completed a questionnaire capturing demographic and lifestyle characteristics, including age, sex, occupation, smoking status, race, and body mass index (BMI). In addition, comorbidity burden was quantified using the Charlson Comorbidity Index (CCI) [14]. The CCI evaluates 19 chronic health conditions-such as myocardial infarction, congestive heart failure, peripheral vascular disease, cerebrovascular disease (excluding hemiplegia), dementia, chronic pulmonary disease, connective tissue disease, peptic ulcer disease, mild liver disease, diabetes (with and without complications), hemiplegia, moderate/severe renal disease, malignancy (non-metastatic and metastatic), leukemia, lymphoma, multiple myeloma, moderate/severe liver disease, and HIV infection. Each condition is assigned a weighted score, and the cumulative total reflects overall comorbidity burden.

#### Symptom outcomes

 Pain intensity at presentation was recorded using a 0–10 visual analog scale (VAS), where 0 indicated no pain and 10 represented the most severe pain imaginable. Pain catastrophizing was measured with the Pain Catastrophizing Scale (PCS), a validated 13-item tool assessing three domains: rumination, magnification, and helplessness [15]. Items were rated from 0 (“not at all”) to 4 (“all the time”), and summed scores represented overall catastrophizing, with higher totals reflecting greater levels [15]. Participant level of function was assessed using the Western Ontario and McMaster Universities Osteoarthritis Index (WOMAC) [16], a validated instrument designed to evaluate osteoarthritis-related pain, stiffness, and functional limitations in daily activities. The instrument demonstrated sound internal consistency (0.93).

#### Behavior and psychosocial determinants

*Total Physical Activity*. This was assessed using a modified Godin Leisure-Time Exercise Questionnaire [17]. Participants indicated whether they engaged in strenuous, moderate, and light exercise, and reported weekly frequency and average session duration for each intensity level. A composite activity score was calculated ((9 x strenuous exercise frequency) + (5 x moderate exercise frequency) + (3 x light exercise frequency).

#### Psychosocial determinants

**Affective Attitudes** toward exercise were assessed using 5 semantic differential items (e.g., enjoyable - miserable, satisfying - annoying), in which lower scores indicated more positive affective responses to physical activity [18]. Cronbach alpha for attitudes scale was found to be 0.93.

**Perceived Social Support**. Perceived social support for exercise was evaluated using an 8-item scale adapted from subjective norm frameworks [19]. Participants rated agreement with statements such as “My family supports me in being physically active” and “I have people who exercise with me or would like to” using a 5-point Likert scale (1 = strongly disagree to 5 = strongly agree). Items also captured support from healthcare providers and social media influence. Cronbach alpha for the support scale was 0.97.

**Perceived Barriers to Exercise** [20]. Barriers to exercise were assessed through a list of items rated from 0 (not a barrier) to 5 (very much a barrier). This included weather, cost of gym membership, transportation to exercise facilities, neighborhood safety, facility access, and presence of sidewalks. Additional dichotomous variables assessed the presence of health-related limitations (e.g., joint pain, asthma, mobility limitations, fear of falling).

**Exercise Self-Efficacy** [21]. Exercise self-efficacy was measured using an 8-item scale asking participants how confident they were in exercising under challenging conditions (e.g., while tired, under stress, lacking social support). Responses were captured on a 5-point scale ranging from “not certain at all” to “very certain.” The internal consistency for this scale was 0.98.

**Exercise Intention** [18]. Intention to exercise was assessed using a validated item measure aligned with the Theory of Planned Behavior. The item was worded; I intend to engage in at least 150 minutes of moderate-to-vigorous intensity/week over the next two weeks”.

### Analysis

All statistical analyses were conducted using R (version 4.3.1; R Foundation for Statistical Computing, Vienna, Austria). This cross-sectional observational study was reported in accordance with the STROBE (Strengthening the Reporting of Observational Studies in Epidemiology) guidelines, as recommended by the EQUATOR Network for observational epidemiological research [22]. The completed STROBE checklist is provided as Supplementary File 1. Descriptive statistics were first used to summarize participant characteristics, including age, sex, ethnicity, body mass index (BMI), Charlson Comorbidity Index (CCI), and education level. Continuous variables were reported as means and standard deviations (SD), and categorical variables were summarized as frequencies and percentages to characterize the sample distribution. Missing data were handled via listwise deletion within each model. Of the 820 enrolled participants, 736 had complete data on the primary study variables and were included in the main analyses. Variable-level missingness was as follows: PCS (*n* = 19 missing; *n* = 801 complete), WOMAC (*n* = 24 missing; *n* = 796 complete), sex (*n* = 6 missing), age (*n* = 8 missing), and BMI (*n* = 12 missing). Sample sizes contributing to each regression model are reported in the corresponding tables. The primary analysis was the multivariable regression model predicting physical activity score from symptom variables (VAS, PCS, WOMAC). All other regression models and sex-based comparisons are designated as secondary or exploratory analyses and should be interpreted with appropriate caution pending replication. Given the number of models tested, formal multiple comparison corrections were not applied; this limitation is acknowledged.

**Fig. 1:**
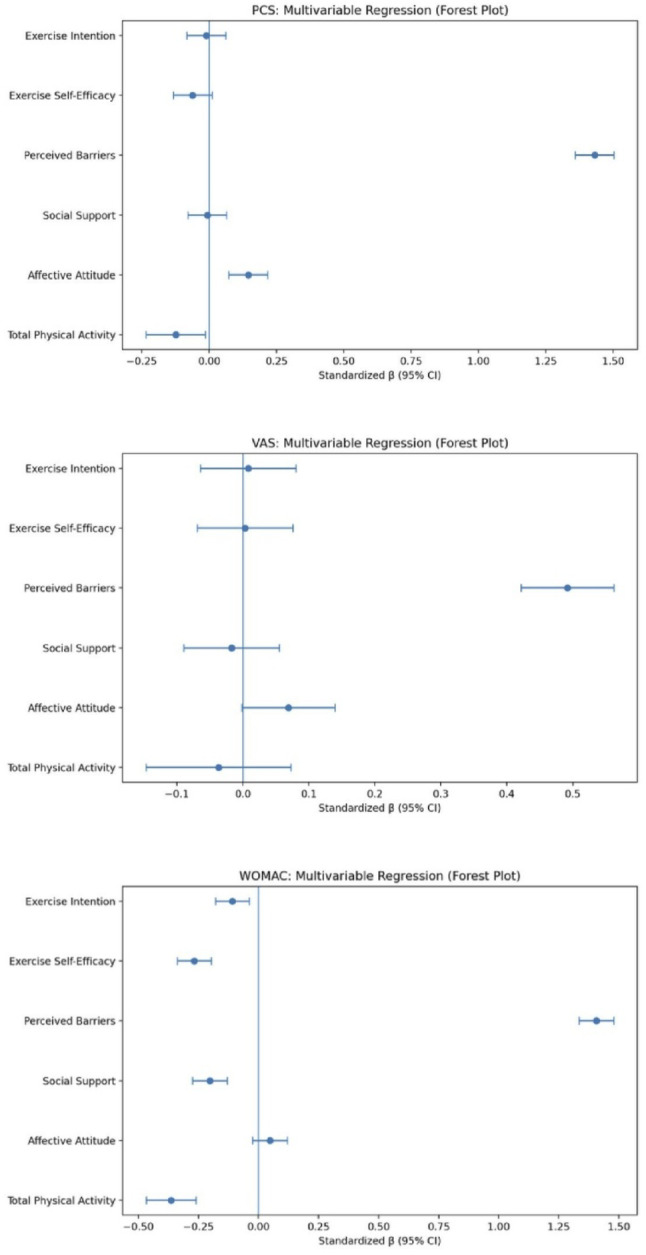
Standardized beta coefficients from multivariable linear regression models examining associations between exercise-related behavioral and psychosocial variables and knee osteoarthritis symptom outcomes. Panels display results for pain intensity (VAS), pain catastrophizing (PCS), and functional limitation (WOMAC). Each effect size represents the independent association between the predictor and the outcome after adjustment for all other exercise-related psychosocial variables included in the model. *VAS* visual analog scale; *PCS* Pain Catastrophizing Scale; *WOMAC* Western Ontario and McMaster Universities Osteoarthritis Index; *PA* Physical activity.

The first set of regressions tested if intention predicted PA, and if proposed behavior determinants predicted intention. To assess the influence of symptom burden on behavioral determinants, WOMAC, VAS, and PCS were simultaneously entered as predictors of perceived barriers, affective attitude, and exercise self-efficacy in three separate models. Impact of clinical symptoms was investigated by testing WOMAC, VAS and PCS as dependent variables on separate models dependent variables as affective attitudes, perceived barriers, exercise self-efficacy. Given that social support may mitigate clinical symptoms, social support was tested across three separate models to identify its potential associations with WOMAC, VAS, and PCS scores Fig. 1.

**Fig. 2:**
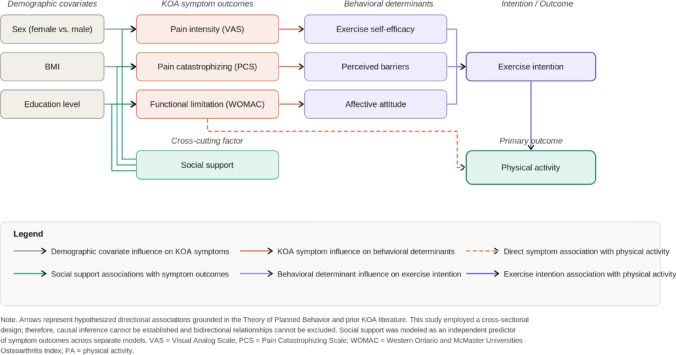
Conceptual model of hypothesized relationships among demographic factors, knee osteoarthritis symptom outcomes, psychosocial behavioral determinants, exercise intention, and physical activity behavior. Arrows represent hypothesized directional associations grounded in the Theory of Planned Behavior and prior KOA literature. Social support was modeled as an independent predictor of all three symptom outcomes across separate regression models. The cross-sectional design of this study precludes causal inference, and bidirectional relationships cannot be excluded. *VAS* Visual Analog Scale; *PCS* Pain Catastrophizing Scale; *WOMAC* Western Ontario and McMaster Universities Osteoarthritis Index; *PA* Physical activity.

The secondary objective was to assess sex-based differences in exercise-related psychosocial variables to determine whether patterns of physical activity engagement and related attitudes differed between male and female patients. Sex-based differences in exercise-related variables (physical activity, affective attitude, perceived barriers, social support, exercise self-efficacy, and exercise intention) were examined using one-way analysis of variance (ANOVA). When significant main effects were observed, post hoc comparisons were conducted using independent samples t-tests. To provide a practical interpretation of between-group differences, Common Language Effect Sizes (CLES) were calculated along with 95% confidence intervals for each comparison [23]. All tests were two-tailed and statistical significance was defined as *p* <.05. Data preparation and statistical analyses were conducted using JMP Pro version 17.0.0. The hypothesized relationships among these constructs are illustrated in Fig. 2.

## Results

### Participant characteristics

A total of 736 patients with symptomatic KOA were included in the study. The mean age was 56.4 ± 11.9 years, and the sample was female (52.2%) and male (47.8%). The majority identified as Hispanic/Latino (59.9%), followed by non-Hispanic White (26.5%) and non-Hispanic Black (9.2%). Average BMI was 29.05 ± 6.29. Kellgren-Lawrence grades were most frequently 1 (*n* = 184) or 2 (*n* = 148). The average Charlson Comorbidity Index (CCI) was 1.88 ± 1.92, and the mean education level was 4.47 ± 1.42 on a 7-point scale. These demographic and clinical characteristics are summarized in Table 1.

**Table 1:** Demographic Characteristics

Characteristic	Total (*n* = 736)
Age, mean (SD)	56.4 (± 11.9)
Sex,%	
Male	352 (47.8)
Female	384 (52.2)
Ethnicity, %	
Hispanic/Latino	441 (59.9)
Non-Hispanic White	195 (26.5)
Non-Hispanic Black	68 (9.2)
BMI, mean (SD)	29.1 (± 6.3)
Kellgren–Lawrence Grade, n (%)	
0	20 (2.7)
1	184 (25.0)
2	148 (20.1)
3	87 (11.8)
4	297 (40.4)

Note. *SD:* Standard Deviation; *BMI *: Body Mass Index.

#### Primary objective

##### Exercise behavior and psychosocial determinants: associations with koa symptom outcomes

Multivariable linear regression modeling revealed that higher physical activity (PA) score was associated with lower pain (VAS; β = −0.037, 95% CI [− 0.063, − 0.011], *p* =.004), lower pain catastrophizing (PCS; β = −0.124, *p* =.004), and better self-reported function (WOMAC; β = −0.364, *p* <.001).The following groups the results based on findings of psychosocial determinants across each model. Complete regression model statistics can be found in Table 2. These associations are also illustrated in Fig. 1.

**Table 2:** Multivariate Linear Regressions Between Exercise Variables and KOA Symptom Outcomes

Exercise Variable		PA	AA	SS	SB	ESE	EI
	Mean	17.84 ± 15.77	4.05 ± 6.02	9.97 ± 12.85	0.386 ± 0.85	8.88 ± 13.60	18.99 ± 24.78
VAS (Mean = 4.63 ± 4.64)	β	−0.037	0.069	0.017	0.492	0.003	0.008
	SE	0.056	0.036	0.037	0.036	0.037	0.037
	p-value	0.004	< 0.001	0.042	< 0.001	0.719	0.066
PCS (Mean = 8.97 ± 9.40)	β	−0.124	0.145	−0.007	1.431	−0.061	−0.010
	SE	0.056	0.037	0.037	0.037	0.037	0.037
	p-value	0.004	0.013	0.796	< 0.001	0.017	0.487
WOMAC (Mean = 24.43 ± 18.11)	β	−0.364	0.048	−0.203	1.407	−0.267	−0.109
	SE	0.053	0.037	0.037	0.037	0.036	0.036
	p-value	< 0.001	0.672	< 0.001	0.077	< 0.001	< 0.001

Note. *PA* Physical Activity; *AA* Affective Attitude; *SS* Social Support; *SB * Perceived Barriers; *ESE * Exercise Self-Efficacy; *EI * Exercise Intention; *VAS * Visual Analog Scale; *PCS * Pain Catastrophizing Scale; *WOMAC* Western Ontario and McMaster Universities Osteoarthritis Index; *KOA * Knee Osteoarthritis; *SE * Standard Error Table 3. Sex-Based Differences in Exercise Variables Among Patients with KOA

#### Physical activity behavior and intention

Higher exercise intention was associated with greater PA score (β = 0.36, *p* <.001). In multivariable models predicting exercise intention, higher positive affective attitudes (β = 0.19, *p* =.001), greater social support (β = 0.26, *p* <.001), higher exercise self-efficacy (β = 0.34, *p* <.001), and fewer perceived barriers (β = 0.16, *p* <.002, contributed to stronger intention to exercise Fig. 2.

#### Impact of pain and functionality on physical activity, and behavior determinants

The multivariate regression model revealed that higher PA score was associated with lower pain (VAS; β = −0.037, *p* =.004), lower pain catastrophizing (PCS; β = −0.124, *p* =.004), and better self-reported function (WOMAC; β = −0.364, *p* <.001).

Three sets of regression models where WOMAC, PCS, and VAS were set as independent variables, and each model tested the effects of affective attitudes, exercise self-efficacy, and perceived barriers. Across all models, only WOMAC revealed to be significant across all models which demonstrated worsening of affective attitudes (β = 0.19, *p* =.001), decreasing exercise self-efficacy (β = 0.34, *p* <.001), and increasing perceived barriers (β = 0.16, *p* <.002).

### Secondary objectives

#### Social support on potentially mitigating pain and functionality

In a series of single-predictor models, greater social support was associated with lower WOMAC (β = −0.30, *p* <.001), lower VAS (β = −0.14, *p* =.021), and lower PCS scores (β = −0.18, *p* =.002).

#### Sex-based differences in psychosocial determinants and physical activity behavior

One-way ANOVA revealed that males reported significantly higher physical activity.

(F(1, 289) = 6.03, *p* =.015), exercise self-efficacy (F(1, 733) = 7.36, *p* =.007), exercise intention (F(1, 734) = 4.69, *p* =.031), and social support (F(1, 734) = 4.32, *p* =.038). No significant sex-based differences were found in affective attitude (*p* =.495) or perceived barriers (*p* =.558). Common Language Effect Sizes (CLES) and corresponding 95% confidence intervals are reported in Table 3.

**Table 3:** Sex-Based Differences in Exercise Variables Among Patients with KOA

Exercise Variable	Male Mean ± SD	Female Mean ± SD	Cohen’s d	*p*	F values	CLES	95% CI
PA	19.99 ± 15.63	15.49 ± 15.63	−0.288	0.015	F(1, 289) = 6.03	0.581	[− 0.433, − 0.142]
AA	4.28 ± 6.05	3.98 ± 6.05	−0.05	0.495	F(1, 733) = 0.47	0.514	[− 0.194, 0.095]
SS	11.17 ± 12.86	9.21 ± 12.86	−0.152	0.038	F(1, 734) = 4.32	0.543	[− 0.297, − 0.007]
SB	0.412 ± 0.854	0.375 ± 0.854	−0.043	0.558	F(1, 734) = 0.34	0.512	[− 0.188, 0.101]
ESE	10.46 ± 13.61	7.73 ± 13.61	−0.201	0.007	F(1, 733) = 7.36	0.556	[− 0.346, − 0.056]
EI	21.40 ± 24.8	17.44 ± 24.8	−0.16	0.031	F(1, 734) = 4.69	0.545	[− 0.305, − 0.015]

Note. *PA* Physical Activity; *AA* Affective Attitude; *SS * Social Support; *SB * Perceived Barriers; *ESE * Exercise Self-Efficacy; *EI * Exercise Intention; *SD* Standard Deviation; *CLES * Common Language Effect Size; *CI * Confidence Interval; *KOA* Knee Osteoarthritis

#### Sex associations with VAS, PCS, and WOMAC

Regression models assessing demographic predictors showed that males had lower symptom scores than females across all three outcomes: VAS (4.34 ± 2.83 vs. 4.91 ± 2.83; *p* =.0064), PCS (7.97 ± 9.37 vs. 9.86 ± 9.37; *p* =.0069), and WOMAC (21.58 ± 17.92 vs. 27.01 ± 17.92; *p* <.0001). However, sex differences in VAS were no longer significant after adjustment for covariates (β = 0.004, SE = 0.0089, *p* =.653). Weight (β = 0.0053, *p* =.0285), BMI (β = 0.0556, *p* =.0009), and education level (β = −0.370, *p* =.036) were each significantly associated with VAS. PCS was associated with height (β = −0.1314, *p* =.0250), weight (β = 0.0265, *p* =.001), BMI (β = 0.284, *p* <.0001), and education (β = −1.692, *p* =.0042). For WOMAC, height (β = −0.324, *p* =.0042), weight (β = 0.076, *p* <.0001), BMI (β = 0.7845, *p* <.0001), and education (β = −3.626, *p* =.0003) all showed strong associations. These demographic associations are reported in Table 4.

**Table 4:** Associations Between Demographic Variables and KOA Symptom Measures

Mean	Sex (Male/Female)		Age (yrs)	Height (in.)	Weight (lbs.)	BMI	Education Level	CCI
			56.41 ± 11.87	66.82 ± 5.98	185.03 ± 43.31	29.05 ± 6.29	4.47 ± 1.42	1.88 ± 1.92
VAS	Male: 4.34 ± 2.83 Female: 4.91 ± 2.83 *p* =.006	β	0.004	−0.023	0.005	0.056	−0.370	0.070
		SE	0.009	0.018	0.002	0.017	0.193	0.055
		p	0.653	0.189	0.029	0.001	0.036	0.197
PCS	Male: 7.97 ± 9.37 Female: 9.86 ± 9.37 *p* =.007	β	−0.025	−0.131	0.027	0.284	−1.692	−0.125
		SE	0.030	0.058	0.008	0.056	0.645	0.182
		p	0.395	0.025	0.001	< 0.001	0.004	0.494
WOMAC	Male: 21.58 ± 17.92 Female: 27.0 ± 17.92 *p* <.001	β	0.104	−0.324	0.076	0.785	−3.626	0.357
		SE	0.057	0.113	0.015	0.107	1.230	0.351
		p	0.071	0.004	< 0.001	< 0.001	< 0.001	0.310

Note.* VAS* Visual Analog Scale; *PCS * Pain Catastrophizing Scale; *WOMAC* Western Ontario and McMaster Universities Osteoarthritis Index; *BMI* Body Mass Index; *CCI* Charlson Comorbidity Index; *KOA* Knee Osteoarthritis; *SD* Standard Deviation; *SE* Standard Error

## Discussion

The study revealed physical activity was associated with lower pain (VAS), reduced pain catastrophizing (PCS), and better function (WOMAC). When physical activity and multiple psychosocial determinants were modeled simultaneously, distinct patterns of association emerged across symptom domains. Higher self-efficacy was linked to lower PCS and WOMAC scores; greater social support was associated with reduced VAS and improved WOMAC outcomes; fewer perceived barriers to physical activity demonstrated lower VAS and PCS scores; and negative affective attitudes toward exercise correlated with increased pain scores. Greater intention to engage in physical activity was connected to better WOMAC scores. These findings extend prior work by demonstrating that psychosocial factors do not exert uniform effects across symptoms but instead show outcome-specific associations with pain, pain-related cognition, and functional limitation.

Previous research has largely focused on the benefits of physical activity in reducing pain and improving function in knee OA and is supported by randomized trials and meta-analyses as a cornerstone of conservative management [24]. However, physical activity can increase pain in some patients, especially those with high levels of pain catastrophizing [25], which reflects the role of cognitive factors in shaping symptom experiences. This suggests that the relationship between physical activity and pain is not uniform and may be influenced by broader psychosocial and contextual factors. Among these, prior work has shown self-efficacy and perceived barriers to be particularly important predictors of exercise adherence and outcomes [9]. The literature has confirmed that high self-efficacy correlates with reduced pain and better function in knee OA [26], while perceived barriers reduce exercise adherence [27]. Notably, pain catastrophizing remains a central moderator influencing both symptom severity and physical activity levels [28]. Exercise intention served as a key behavioral correlate of physical activity, with multiple psychosocial determinants independently contributing to intention formation through pathways involving attitudes, perceived barriers, self-efficacy, and social context.

In our cohort, males reported significantly higher physical activity, exercise self-efficacy, social support, and intention to exercise compared to females, despite no sex differences in affective attitude or perceived barriers. Multiple studies indicate that female knee OA patients tend to be less physically active than male patients and face greater psychosocial hurdles to exercise engagement. Women with knee OA often report lower exercise self-efficacy and motivation alongside higher perceived barriers (e.g., health concerns, social expectations) compared to men [26]. They also exhibit elevated levels of negative psychosocial factors such as depressive symptoms and pain catastrophizing relative to males [10, 29]. These disparities have tangible implications for outcomes: women generally experience more severe pain and functional impairment in knee OA than men, and evidence suggests that psychosocial differences may mediate this gap [10]. For example, in a cohort of older adults with knee OA, higher depression in women was linked to their greater pain, with depressive symptoms mediating sex differences in pain intensity [30]. Sex-based differences in psychosocial profiles (confidence, support, affect, and coping) likely contribute to women’s higher pain and disability burden in knee OA and may necessitate gender-tailored interventions [10]. Symptom severity was independently linked to higher perceived barriers, more negative affective attitudes toward exercise, and lower exercise self-efficacy, suggesting a reinforcing cycle.

In our cohort, males reported significantly lower symptom severity across all three outcomes, VAS, PCS, and WOMAC, compared to females. Higher BMI was independently associated with worse outcomes, including increased VAS, PCS, and WOMAC scores, while higher education level was associated with better symptom scores, consistent with prior studies identifying obesity and female sex as strong predictors of knee OA symptom severity and disability [31–33]. By modeling demographic and psychosocial factors concurrently, this study clarifies their relative contributions and highlights that both fixed factors (sex, BMI) and modifiable psychosocial variables (self-efficacy, social support, perceived barriers) independently shape symptom burden. Notably, several behavioral factors are actionable targets for intervention, and prior work has shown that targeting self-efficacy and social support has the potential to improve symptom outcomes in knee OA [26]. Although we are not the first to report associations between individual constructs and knee OA severity, this study adds value by incorporating a large, clinically confirmed cohort from a predominantly Hispanic/Latino population underrepresented in prior research.

Previous work has examined individual psychosocial constructs (e.g., self-efficacy or catastrophizing) in relation to KOA outcomes, but rarely in combination within a single multivariate framework [34]. Our study extends this work by concurrently modeling multiple behavioral determinants, affective attitudes, perceived barriers, social support, self-efficacy, and exercise intention alongside three validated symptom outcomes in a single cohort. Additionally, our sample is drawn from a predominantly Hispanic/Latino population in South Florida, a demographic group underrepresented in prior publications on KOA psychosocial research [35]. Finally, the inclusion of sex-stratified analyses with standardized effect sizes provides a level of methodological detail that complements and extends findings from prior RHEI studies on sex differences in KOA.

### Strengths and limitations

This study has several strengths worth noting. Our sample size (*n* = 736) includes a predominantly Hispanic/Latino population, a group underrepresented in prior KOA psychosocial research. Multiple behavioral determinants were examined concurrently within a single multivariate framework rather than in isolation, and all primary outcomes were assessed using validated instruments. Sex-stratified analyses were conducted with standardized effect sizes and 95% confidence intervals reported, and the study adhered to STROBE guidelines for observational research. The study also has some limitations worth noting. The cross-sectional design precludes causal inference, and bidirectional relationships cannot be excluded, higher symptom burden may itself reduce self-efficacy and social support rather than the reverse. Additionally, sex comparisons in psychosocial variables were primarily unadjusted, and residual confounding by socioeconomic factors cannot be excluded. Assessing related conditions such as kinesiophobia and comorbidities such as depression and anxiety would further allow these factors to be controlled in the analysis and yield greater precision. Lastly, the series of multiple regression models has the potential to increase Type I error. Taken together, a prospective design employing structural equation modeling could be a next logical study to conduct.

The wide spectrum of Kellgren–Lawrence grades (0–4) introduces sample heterogeneity. Subgroup analyses comparing mild-to-moderate (KL grades 0–2, *n* = 351–352) and moderate-to-severe disease (KL grades 3–4, *n* = 130–137, varying by variable due to listwise deletion) revealed that associations between psychosocial determinants and symptom outcomes were broadly consistent across severity strata (Supplementary Table S1). Patients with higher KL grades reported significantly greater functional limitation (WOMAC; *p* =.046, d = − 0.21) and more negative affective attitudes toward exercise (*p* =.005, d = − 0.27), while exercise self-efficacy and other psychosocial variables did not differ significantly between groups. Moderation analyses further indicated that KL grade significantly moderated the associations of social support with VAS (*p* =.037) and WOMAC (*p* =.015), and self-efficacy with WOMAC (*p* =.024), suggesting that radiographic severity may amplify select psychosocial–symptom relationships in more advanced disease (Supplementary Table S2). These subgroup findings should be interpreted cautiously given the reduced sample sizes in the moderate-to-severe group.

## Conclusion

This investigation suggests that KOA symptom severity and physical activity engagement are linked in a complex manner. Symptoms influence physical activity participation, yet symptom burden itself is shaped by both fixed demographic factors such as sex, BMI, and education, and modifiable psychosocial variables including exercise self-efficacy, social support, intention, and perceived barriers. These psychosocial determinants may mediate the relationship between physical activity and symptom outcomes which shows the importance of patients’ behavioral and psychosocial context in pain and disability experiences. Integrating psychological interventions with structured exercise may offer a more effective and personalized approach to managing symptoms of KOA.

## Supplementary Information

Below is the link to the electronic supplementary material.

Supplementary Material 1

## Data Availability

The data that support the findings of this study are available from the corresponding author upon reasonable request.

## Declarations

### Disclaimer

The authors declare that no part of this manuscript has been published or is currently under consideration for publication elsewhere, in whole or in part.

### Related congress abstract

No congress abstract from this study has been previously published. The authors confirm there is no prior related abstract publication associated with this manuscript.
