# Supplementary material for: Sex differences in physical activity, psychosocial determinants, and symptom outcomes in knee osteoarthritis: a cross-sectional analysis of a Hispanic/Latino-predominant cohort
Source: Rheumatol Int. 2026 May 23;46(6):102. doi: 10.1007/s00296-026-06148-7 (PMC13198481; doi:10.1007/s00296-026-06148-7)
Supplement: Supplementary file 1 — Supplementary Material 1 [file 296_2026_6148_MOESM1_ESM.docx]

**Supplementary Table S1.** Subgroup Comparisons of Symptom Outcomes, Physical Activity, and Psychosocial Determinants by Kellgren–Lawrence (KL) Grade

| **Variable** | **Mild–Moderate (KL 0–2)** | | **Moderate–Severe (KL 3–4)** | | **t** | **p** | **Cohen's d [95% CI]** |
| --- | --- | --- | --- | --- | --- | --- | --- |
|  | **n** | **M (SD)** | **n** | **M (SD)** |  |  |  |
| Pain Intensity (VAS) | 351 | 4.50 (2.74) | 137 | 4.80 (2.92) | −1.07 | .286 | −0.11 [−0.30, 0.09] |
| Pain Catastrophizing (PCS) | 348 | 8.35 (8.48) | 136 | 9.61 (9.51) | −1.42 | .157 | −0.14 [−0.34, 0.06] |
| Functional Limitation (WOMAC) | 338 | 23.20 (16.65) | 130 | 26.68 (17.43) | −2.00 | **.046*** | **−0.21 [−0.41, −0.00]** |
| Physical Activity Score | 186 | 20.02 (21.88) | 84 | 16.08 (14.71) | 1.50 | .134 | 0.21 [−0.05, 0.47] |
| Affective Attitude | 351 | 5.26 (6.12) | 137 | 7.07 (7.09) | −2.80 | **.005**** | **−0.27 [−0.47, −0.07]** |
| Social Support | 352 | 13.65 (13.44) | 137 | 15.50 (12.74) | −1.39 | .166 | −0.14 [−0.34, 0.06] |
| Perceived Barriers | 352 | 0.53 (0.99) | 137 | 0.58 (0.88) | −0.44 | .659 | −0.05 [−0.24, 0.15] |
| Exercise Self-Efficacy | 351 | 12.73 (15.18) | 137 | 12.88 (13.87) | −0.10 | .923 | −0.01 [−0.21, 0.19] |
| Exercise Intention | 352 | 26.33 (26.11) | 137 | 29.07 (24.74) | −1.06 | .292 | −0.11 [−0.30, 0.09] |

**Note.** KL = Kellgren–Lawrence classification grade. Mild–Moderate group includes KL grades 0–2; Moderate–Severe group includes KL grades 3–4. M = mean; SD = standard deviation. Cohen's d was calculated using pooled standard deviation; 95% confidence intervals are approximate. Affective Attitude: lower scores indicate more favorable attitudes toward exercise. All other psychosocial variables: higher scores indicate more of the construct (greater support, more barriers, higher self-efficacy, stronger intention). Physical Activity Score = Godin Leisure-Time Exercise Questionnaire composite score (dimensionless weighted frequency index; higher = more active).

** p < .05 ** p < .01 (two-tailed independent samples t-tests; missing data handled via listwise deletion per variable).*

**Supplementary Table S2.** Moderation of Psychosocial–Symptom Associations by Kellgren–Lawrence (KL) Grade: Interaction Term Regression Results

| **Outcome** | **Interaction Term** | **n** | **β** | **SE** | **t** | **p** | **95% CI** |
| --- | --- | --- | --- | --- | --- | --- | --- |
| **Pain Intensity (VAS)** | KL × Affective Attitude | 488 | −0.023 | 0.017 | −1.36 | .176 | [−0.057, 0.010] |
|  | KL × Social Support | 488 | **−0.019** | 0.009 | −2.09 | **.037** | **[−0.037, −0.001]** |
|  | KL × Perceived Barriers | 488 | 0.054 | 0.128 | 0.42 | .674 | [−0.197, 0.305] |
|  | KL × Exercise Self-Efficacy | 488 | −0.017 | 0.009 | −1.94 | .053 | [−0.034, 0.000] |
| **Pain Catastrophizing (PCS)** | KL × Affective Attitude | 484 | −0.066 | 0.055 | −1.19 | .236 | [−0.174, 0.043] |
|  | KL × Social Support | 484 | −0.052 | 0.029 | −1.81 | .071 | [−0.109, 0.004] |
|  | KL × Perceived Barriers | 484 | −0.577 | 0.407 | −1.42 | .157 | [−1.374, 0.220] |
|  | KL × Exercise Self-Efficacy | 484 | −0.044 | 0.027 | −1.62 | .105 | [−0.097, 0.009] |
| **Functional Limitation (WOMAC)** | KL × Affective Attitude | 485 | −0.089 | 0.108 | −0.83 | .409 | [−0.301, 0.123] |
|  | KL × Social Support | 485 | **−0.135** | 0.055 | −2.44 | **.015** | **[−0.243, −0.027]** |
|  | KL × Perceived Barriers | 485 | −0.123 | 0.800 | −0.15 | .878 | [−1.691, 1.445] |
|  | KL × Exercise Self-Efficacy | 485 | **−0.117** | 0.051 | −2.27 | **.024** | **[−0.218, −0.016]** |

**Note.** Each row represents the interaction term (KL grade × psychosocial predictor) from a separate regression model containing: intercept + KL grade (centered) + psychosocial predictor (centered) + interaction term. KL grade was treated as a continuous variable (0–4). All predictors were mean-centered prior to computing interaction terms to reduce multicollinearity. β = unstandardized regression coefficient for the interaction term; SE = standard error; 95% CI = confidence interval for β. Significant interaction terms (highlighted in red) indicate that the strength of the psychosocial–symptom association varied as a function of radiographic severity. Probing of significant interactions revealed that all effects were in the same direction at low and high KL grades (no crossover); the Social Support → VAS association was weaker at higher KL grades, while the Social Support → WOMAC and Self-Efficacy → WOMAC associations were stronger at higher KL grades. n = listwise complete cases per model.

** p < .05 (two-tailed). No corrections for multiple comparisons were applied; findings should be considered exploratory.*
